# Acceptability of Circle of Security-Parenting groups in NHS community perinatal mental health services in England: parent and practitioner perspectives

**DOI:** 10.1186/s40359-026-04068-6

**Published:** 2026-02-07

**Authors:** Zoë Darwin, Lani Richards, Amy Clarke, Kavita Trevena, Sophia Nahz Rehman, Jude Field, Nina Morris, Innamana Pettyll, Basanti Aryal, Kim Alyousefi-van Dijk, Ruth O’Shaughnessy, Nic Horley, Ed Waddingham, Daphne Babalis, Victoria Cornelius, Pasco Fearon, Steve Pilling, Jiunn Wang, Elena Pizzo, Peter Fonagy, Camilla Rosan

**Affiliations:** 1 University of Huddersfield, Huddersfield, UK; 2 Anna Freud, London, UK; 3 Mersey Care NHS Foundation Trust, Prescot, UK; 4 University College London, London, UK; 5 West London NHS Trust, Southall, UK; 6 Imperial Clinical Trials Unit, Imperial College London, London, UK; 7 Cambridge University, Cambridge, UK

**Keywords:** Acceptability, Circle of security, Infant mental health, Perinatal mental health, Qualitative

## Abstract

**Background:**

Perinatal mental health (PMH) difficulties are prevalent and often accompanied by parent-infant relationship difficulties. National Health Service community PMH services (PMHS) support birthing parents (typically mothers) experiencing moderate-to-severe and complex mental health difficulties. While PMHS primarily address maternal mental health, treatment can include interventions targeting parent-infant relationships. The Circle of Security-Parenting (COS-P) programme is widely used within PMHS in England and offers a potential solution to the evidence gaps for interventions that: i) target both parental mental health and parent-infant relationship quality; (ii) are transdiagnostic; and iii) delivered in groups. This study evaluates the acceptability of COS-P, an attachment-informed, group intervention delivered in PMHS in ten 90-min sessions, predominantly online.

**Methods:**

This qualitative study analysed the perspectives of parents (COS-P recipients) and practitioners (COS-P providers) in the intervention arm of a wider randomised controlled trial. Data collection involved interviews (58 parents, 7 practitioners) and focus groups (6 practitioners). Reflexive thematic analysis was conducted by a team including co-researchers with lived experience and interdisciplinary academics and practitioners.

**Results:**

Four themes were constructed: (1) ‘Flamingos’, capturing the power of the group in normalising and validating demands relating to motherhood and PMH; (2) ‘Practise Babies’, highlighting the universal necessity and benefit of practising relationship skills, without expectations of perfection and with opportunities for repair; (3) ‘the Dark Things’, describing the emotional intensity for parents and practitioners arising from current and past relationships, occasionally necessitating extra support; and (4) ‘the Ripples’, illustrating shifts in understanding and compassion that may extend beyond the parent-infant relationship and interact with other interventions. These themes encompass both positive and negative experiences for parents and practitioners, as well as practical considerations for implementing COS-P within PMHS.

**Conclusions:**

Although COS-P is positively regarded by many parents and practitioners in PMHS, attention to individual and service-specific factors remains crucial. Findings underscore the importance of trauma-informed approaches, particularly regarding intervention timing, sequencing, and ensuring personal agency in treatment decisions. Moreover, the effective facilitation of parent-infant psychological group interventions demands significant skill and resource allocation before, during, and outside sessions, impacting workforce planning, practitioner training, and supervision.

**Trial registration:**

ISRCTN18308962. Registered 18/02/2022.

**Supplementary Information:**

The online version contains supplementary material available at 10.1186/s40359-026-04068-6.

## Background

Perinatal mental health (PMH) difficulties encompass conditions with onset, relapse, or exacerbation during pregnancy and the postnatal period. These difficulties are prevalent and can be experienced by birthing parents (i.e. mothers, and trans and gender diverse parents) and non-birthing parents (i.e. fathers and other co-parents). Approximately one in four birthing parents experience PMH difficulties in England, with higher prevalence likely in low- and middle-income countries [19, 20]. Although adverse impacts of PMH difficulties are not inevitable, timely intervention can substantially reduce negative consequences for family members, including infants and their siblings. Economic analyses emphasise significant transgenerational impacts, supporting increased investment in the timely identification and management of PMH conditions [2]. In England, this has driven rapid expansion of specialist NHS community perinatal mental health services (PMHS). These services cater for the approximately 10% of birthing parents who have moderate-to-severe or complex mental health needs and therefore require a more intensive and specialist provision than can be provided in primary care [15]. Equivalent services do not exist for non-birthing parents.

PMHS primarily assess and treat maternal mental health, though they also evaluate the parent-infant attachment relationship [39]. Crucially, evidence gaps persist for interventions that are: (i) transdiagnostic across PMH conditions; (ii) focused on both parental mental health and parent-infant relationship quality; and (iii) deliverable in a group setting, thus necessitating further investigation [37]. Group interventions are increasingly attractive in mental health services as an efficient solution to increased demands on services [42], additionally, in the perinatal context, peer support may normalise difficulties and diminish isolation [32]. A meta-synthesis on parenting programmes found that aspects perceived to be important by parents include the practitioner facilitating the group, the value of the group, and programme content [6]. The review additionally identified challenges for practitioners in balancing flexibility to meet individuals’ needs with maintaining programme fidelity, and the need for further research on parents’ and practitioners’ perspectives of specific programmes to ensure programme success. The Circle of Security-Parenting programme (COS-P; [36]) was identified as an attachment-focused group intervention that has potential to address the identified evidence gaps. Additionally, a 2016 meta-analysis on clinical efficacy of COS-P indicated possible gains for maternal psychopathology, parental self-efficacy and child attachment security [45]. However, most studies contributing to these findings up until that point included small sample sizes and uncontrolled designs, warranting a more rigorous evaluation. Since commencement of this research, the NHS has significantly invested in COS-P training within PMHS from 2019 to 2024 [33]. This mirrors broader international trends observed in Australia and Europe, where public investment and dissemination of COS-P have exceeded its established evidence base [14, 16, 27] and highlights the timeliness of this research.

Previous qualitative research on the acceptability of COS-P has been reported as encouraging in small-scale studies. However, while COS-P was designed for caregivers of children from around four months to six years, the existing acceptability evidence relates primarily to contexts involving pre-school or primary school-aged children, warranting in-depth research with parents of infants. For instance, interviews with 12 mothers in a Norwegian adult public health setting where COS-P was offered universally to parents indicated positive experiences when delivered alongside ongoing outpatient psychotherapy; notably, providers extended delivery from eight to 12 sessions to accommodate participants’ needs related to childhood trauma and neglect [16]. Similarly, analysis of interviews in Ireland with eight mothers and one father, whose index children were aged 4–9 years, reported parents’ “immense satisfaction” with COS-P delivered through various Child and Family services [14].

Researchers recommend gathering both the perspectives of parents and practitioners when evaluating parenting programmes to ensure consideration of recipients and providers [31]. Existing COS-P acceptability evidence has focused on parent perspectives. Notably, in the one study that integrated both parent and practitioner perspectives [28], findings were more mixed compared to studies involving parents only. Specifically, Australian research with 14 parents (with infants and young children aged 0–6 years and 20 COS-P practitioners across diverse service contexts and varied delivery formats found that COS-P was perceived as “effective, relevant and accessible for a broad range of parents … [but] insufficient or unsuitable for some parents” (p.453, highlighting the necessity for careful consideration in contexts identified as high-risk within early parenting services [28]. Additionally, although this paper featured some parents of infants, analysis did not consider the child’s age and furthermore, with one exception, these parents were additionally caring for older children, which may limit transferability to first-time parents.

Alongside gaps concerning parents of infants, and integration with practitioner perspectives, gaps exist concerning delivery mode. Existing evidence predominantly concerns in-person COS-P delivery (e.g., [14, 16, 28]), limiting transferability to contemporary clinical ways of working. Currently, insights into remote delivery are limited to Cook et al. [7], reflecting on one remotely delivered COS-P group within the COVID-19 pandemic, positioning online delivery as convenient and engaging but advocating for further research exploring suitability for different populations.

Indeed, population context is crucial when evaluating complex interventions such as COS-P [41]. The current study was needed because learning from existing evidence may not directly transfer to specialist PMHS, where parents typically experience moderate-to-severe and complex PMH difficulties and are caregivers to infants. The Circle of Security Intervention (COSI) trial was therefore conducted within specialist community PMHS in England [37, 38]. The primary aim of the trial was to evaluate the clinical effectiveness of COS-P plus treatment as usual, compared to treatment as usual alone. The secondary aim – reported here—was to explore the acceptability of COS-P from the perspectives of parents (COS-P recipients) and practitioners (COS-P providers) within NHS community PMHS in England, where acceptability can be understood as “the extent to which people delivering or receiving a healthcare intervention consider it to be appropriate, based on anticipated or experienced cognitive and emotional responses to the intervention” [40].

## Methods

**Fig. 1:**
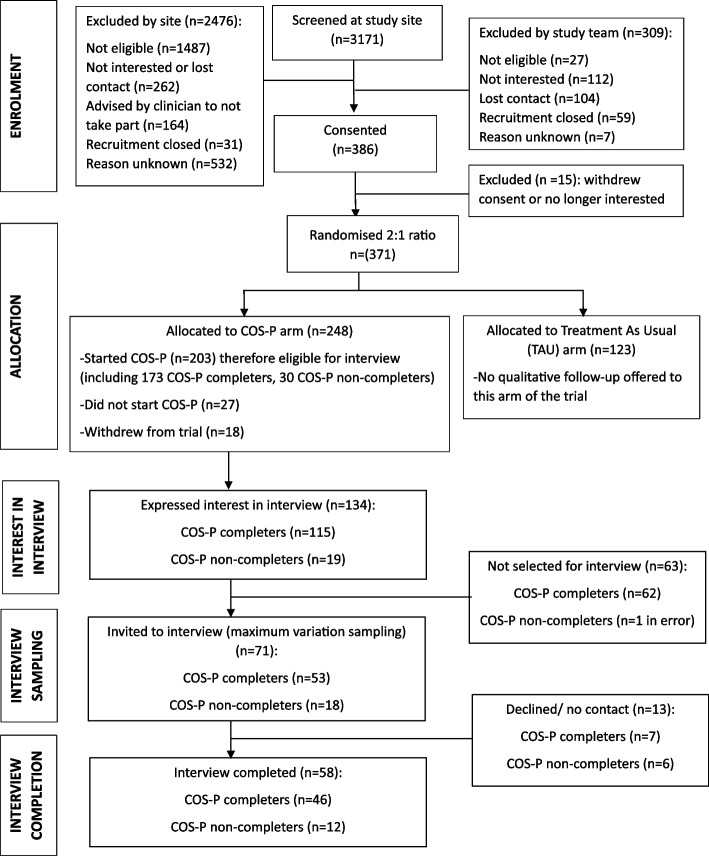
Parent participant flow

This qualitative study was embedded within the broader COSI trial, a multicentre, parallel-arm, randomised controlled trial where parent participants were randomised in a 2:1 ratio to COS-P plus treatment as usual or to treatment as usual alone. A total of 371 birthing parents were recruited for the main trial between January 2022 and October 2023. As shown in Fig. 1, 248 participants were randomised to COS-P. As per the overall trial’s inclusion criteria, all participants received care from one of the 10 NHS PMHS affiliated with the trial for moderate-to-severe, or complex, psychopathology as indicated by an average item score of 1.1 or more on the Clinical Outcomes in Routine Evaluation–Outcome Measure (CORE–OM; [10]) and difficulties in the parent-infant bond as indicated by a total score of 12 or more on the Postpartum Bonding Questionnaire (PBQ; [5]). Additionally, all participants were aged at least 18 years, able to give consent, had an infant under 12 months of age with no significant illness or developmental disorder, were able to attend COS-P sessions without being under the influence of substances and had not received COS-P before. An exclusion criterion of not having conversational levels of English was used initially, but this was later removed to widen access. Lastly, a total of 24 practitioners from the 10 affiliated NHS PMHS were recruited to the trial. All were not previously trained in COS-P but had experience in delivering psychological therapies, group facilitation and/or parent-infant focussed support. Twenty-one practitioners delivered 51 groups across these sites within the main trial. Clinical effectiveness outcomes are reported separately [38], where all details are provided to show adherence to the Consolidated Standards of Reporting Trials (CONSORT) [18]. No changes were made to the published protocol [37]. Qualitative data was collected between July 2022 and April 2024. Parents, practitioners and qualitative researchers were not blinded to treatment allocation. Supplementary File 1 summarises which CONSORT items are provided in this paper, consistent with journal requirements. The focus of this paper is the perspectives of COS-P recipients and providers and therefore only relates to the intervention arm. This paper is reported following Standards for Reporting Qualitative Research (SRQR) [34].

### Intervention description

COS-P is a manualised intervention comprising eight modules informed by psychoeducational, cognitive-behavioural, attachment, and psychodynamic theories. The intervention utilises facilitated observation and guided reflection on existing video footage of parent–child interactions, supplemented by visual resources and guided reflection. As detailed in the study protocol [37], this research involved a 10-session perinatal adaptation of COS-P, facilitated by a trained lead practitioner and supported by a co-facilitator without formal COS-P training; babies were welcome to attend sessions. Perinatal adaptation to the manual involved: extending to 10 sessions to allow more time, providing prompts and reflection questions to support practitioners to focus on the concerns of parents of very young infants, and adding images of babies to some of the diagrams used in the resources. Recruitment and intervention sites (i.e., NHS PMHS) were chosen based on being new to COS-P and not having any staff trained in the intervention prior to the trial. Practitioners received the standard 24 h of online training within one week, supplemented by a 1.5 h workshop focused on perinatal adaptations, plus 20 h of coaching supervision, all provided by COS International, the intervention developers. After completion of training, all practitioners were encouraged to conduct an initial ‘practise group’ prior to trial delivery. The intervention developers were not involved in study design, data collection, data analysis, data interpretation, or writing of the manuscript. The intervention was delivered primarily online, reflecting evolving post-pandemic healthcare practices.

### Data collection

Individual interviews were chosen for use with parent participants, due to the sensitive nature of the discussions, to reduce social pressures (when expressing experiences of a group intervention in a mental health context) and reduce time burden on parents. Originally, data collection with practitioners was planned to solely use focus groups, given established usefulness for exploring shared and differing perspectives; however, clinical commitments and work patterns presented practical challenges, resulting in additionally offering individual interviews. It was considered appropriate to include data both from parents and practitioners, and across interviews and focus groups, within a single dataset, as the analysis aimed to explore overarching patterns of meaning, while remaining attentive to differences and to the context in which the data was produced [4]. The interview and focus group topic guides were developed in collaboration with lived experience co-researchers to examine acceptability (reported here) and wider barriers and facilitators to access (unpublished currently). The guides are available in the protocol [37]. The interviews were conducted by an experienced qualitative midwife-researcher (JF) with interests in trauma, who was new to COS-P and to the specialist clinical context though bringing experience with wider PMH needs, she also led the focus groups, which were additionally attended by an experienced PMH researcher (ZD) and lived experience co-researcher (LR).

### Recruitment and participant flow

**Fig. 2:**
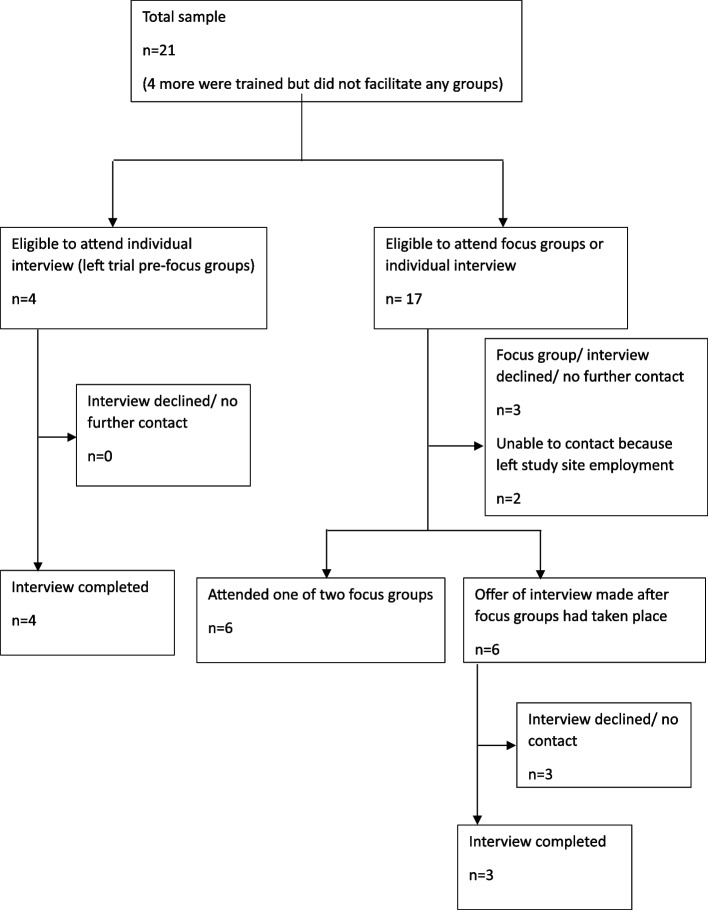
Practitioner participant flow

Interview and focus group participants were recruited from the intervention arm of the main COSI trial after completion of the intervention and the 3-month follow up timepoint which included quantitative measures (see [38]). The paper reporting clinical effectiveness findings [38] provides full details on recruitment of the 371 main trial participants who were recruited between January 2022 and October 2023. Qualitative data was collected between July 2022 and April 2024. As shown in Fig. 1, 248 participants were randomised to COS-P; this involved 51 groups across 10 NHS sites in England. As shown in Fig. 1, out of the 248 participants randomised to receive the intervention, 203 began COS-P and were therefore eligible to be interviewed about their COS-P experiences. Participants were able to indicate their interest in being interviewed either through an online survey link, or by email, text message or telephone contact with the qualitative researcher. Approximately two-thirds (i.e., 134) indicated interest. All COS-P non-completers (those attending 1–5 group sessions) and a purposive subsample of COS-P completers (attending 6 or more sessions) were invited to interview (see Fig. 1). Maximum variation sampling was used (Sandelowski, 1995) to determine interview invitation, concerning participant characteristics (including ethnicity, family composition (e.g. first-time and subsequent parents), infant age, and parent relationship status), study site, recruitment block, number of sessions completed, and group size. This approach resulted in 58 parent interviews (see Fig. 1). Additionally, all 21 practitioners who facilitated at least one COS-P group were invited to participate in either interviews or focus groups, yielding seven individual interviews and six practitioners attending one of two focus group (total 13 participants; see Fig. 2).

### Sample characteristics

As shown in Table 1, 74% of the sample identified as White British and 90% of the birthing parents identified as women. The sample included first-time and subsequent parents, with 59% having more than one child in the household. The mean infant age was 20.8 weeks at baseline (SD 12.6). Parents typically reported multiple mental health difficulties leading to their PMHS referral, with most identifying depression (83%) and anxiety (90%); 95% reported a previous history of mental health difficulties. Practitioner participants comprised Practitioner Psychologists (Clinical/Counselling, *n* = 10) and Mental Health Nurses (*n* = 3). Most practitioners (11/13) had prior clinical experience in parent-infant work, and the majority (12/13) had previously facilitated therapeutic groups, though only four had experience of online facilitation. At data collection, excluding ‘practise groups’, eight practitioners had delivered three or more trial groups.

**Table 1:** Parent sample characteristics (*n* = 58)

*Variable*	*mean (sd) for continuous variables, n (%) for categorical variables*
Age (years)	31.9 (5.0)
Ethnicity	
White British	43 (74%)
White other	7 (12%)
Other	5 (9%)
Missing	3 (5%)
Relationship status	
Single	5 (9%)
In a relationship (living together)	48 (83%)
In a relationship (not living together)	1 (2%)
Not known	2 (3%)
Missing	2 (3%)
Sexual orientation	
Straight	47 (81%)
Other	10 (17%)
Missing	1 (2%)
Gender	
Woman	52 (90%)
Man (including trans man)	0 (0%)
Non-binary	3 (5%)
Other	1 (2%)
Missing	2 (3%)
Mental health difficulties leading to referral to PMHS	
Depression	48 (83%)
OCD	8 (14%)
Anxiety	52 (90%)
Personality difficulties	6 (10%)
Trauma	24 (41%)
Psychosis	4 (7%)
Bi-polar disorder	2 (3%)
Other	4 (7%)
Previous mental health difficulties	
Yes	55 (95%)
No	2 (3%)
Prefer not to say	0 (0%)
Missing	1 (2%)
Income above deprivation threshold	
Yes	22 (38%)
No	34 (59%)
Missing	2 (3%)
Highest completed level of education	
Primary education or less	1 (2%)
Secondary education	5 (9%)
Tertiary/further education (e.g., college)	18 (31%)
Higher education (e.g., University degree)	31 (53%)
Other general education	1 (2%)
Prefer not to say	0 (0%)
Missing	2 (3%)
Housing	
Homeowner	33 (57%)
Other	22 (38%)
Missing	3 (5%)
Employment status	
Employed or self-employed	39 (67%)
Unemployed or in education/training	17 (29%)
Missing	2 (3%)
Religion	
Christian	19 (33%)
None	31 (53%)
Other	2 (3%)
Prefer not to say	5 (9%)
Missing	1 (2%)
Country of birth	
United Kingdom	47 (81%)
Elsewhere	7 (12%)
Missing	4 (7%)
First language	
English	50 (86%)
Other language (but having good knowledge of English)	6 (10%)
Missing	2 (3%)
Child age (in weeks)	20.8 (12.6)
Child first born status (measured as having more than one < 18-year-old in the household)	
First born	24 (41%)
Not first born	34 (59%)
Number of previous pregnancies	
0	17 (29%)
1	11 (19%)
> 1	30 (52%)
Child sex	
Female	31 (53%)
Male	26 (45%)
Missing	1 (2%)
CTQ score at baseline	48.1 (20.2)
CORE-OM score at baseline	67.7 (20.6)
CORE-OM score at 3 m	56.7 (18.2)
PBQ score at baseline	37.8 (17.3)
PBQ score at 3 m	27.0 (14.0)

CORE-OM = Clinical Outcomes in Routine Evaluation [10], CTQ = Childhood Trauma Questionnaire [3], PBQ = Postpartum Bonding Questionnaire [5]

### Analysis

Interviews and focus groups were audio-recorded, transcribed verbatim, and imported into NVivo 14 [25] to support analysis using reflexive thematic analysis [4]. The lead analysts (ZD, JF) engaged in ongoing familiarisation during data collection, including regular discussions to reflect on emerging ideas, assess data quality [17], and consider information power [26]. Formal analysis began by the lead analysts collaboratively coding a subset of transcripts, supporting exploration of initial impressions and developing early coding ideas to provide starting points for thinking with lived-experience co-researchers about meaning in the data. Elements of the data were brought by the lead analysts (ZD, JF) to a series of seven collaborative meetings with lived-experience co-researchers (LR, AC, KT, SNR). These meetings focused on specific aspects (e.g. experiences relating to being in a COS-P group, the role of the practitioner, support needs when taking part in COS-P) and brought in data from a wider range of transcripts, as part of the iterative nature of the work. All colleagues contributed to coding and took part in interpretive discussions and collective reflection about divergence in coding, iteratively developing patterns of meaning. These meetings spanned a two-year period, taking place approximately every three months, and were typically arranged as half-day meetings, with accompanying preparatory activities. Additionally, academic team members (JF, BA, LR, NM, IP) worked with the dataset to ensure that all the data was considered beyond what was feasible within the seven meetings, with regular reflective exchanges with ZD through written exchanges and online meetings. ZD led the development of central organising concepts for themes and sub-themes. Consistent with reflexive thematic analysis being interpretivist, these were constructed iteratively and refined through discussions with lived-experience co-researchers (LR, AC, KT, SNR), other research assistants involved in coding (JF, BA, NM, IP), and clinical trial team members (CR, ROS, NH), with consideration of how individual perspectives - as parents and as perinatal researchers and practitioners - may be shaping the analysis. We used naming quotes for each theme to strongly illustrate a central idea using parent participants’ own words, while sub-theme names (created in our own words) conveyed our key message of each sub-theme and captured nuance in the broader theme. Once we had our candidate themes, team members (ZD, BA, NM, IP) then recursively revisited coding across the dataset to check alignment with the themes’ organising concepts, revising codes and themes as needed. Final thematic structures and language were further shaped by collaborative discussions at a dedicated trial team away day. Consistent with reflexive thematic analysis [4], we continued to evolve our themes during manuscript writing. Additional revisions were made in response to the peer review process, while ensuring we did not depart from the themes constructed by the analysis team. We used a team approach to select illustrative quotes for the manuscript and determine their length.

### Reflexive statement for those involved with the analysis

Amongst the lived experience co-researchers, the team included one member who had received COS-P in-person and individuals who had received other group-based psychological interventions within PMHS, including some online. The academic qualitative researchers included birthing and non-birthing parents. One of the lived experience co-researchers had shadowed COS-P in a professional role. Additionally, practitioners in the wider study team included individuals with experience of in-person and online delivery of group-based psychological interventions within this clinical context, including one specific to COS-P. We note parallels with elements of our experiences in working together, where our meetings were predominantly online with some in-person, and sometimes with infants in attendance. We note too that our research team, parent participants and practitioner participants were predominantly women, which mirrors this clinical context.

## Results

We constructed four themes, each named using a parent quote: Flamingos; Practise Babies; the Dark Things; and the Ripples. These four themes illustrate what mattered to parents and practitioners in evaluating COS-P’s acceptability. The theme of Flamingos captures the power of the group and how participants are nurtured through COS-P content, peer interactions and practitioner skills. The theme of Practise Babies emphasises the value of having permission to be a learner when practising relationship skills. The Dark Things theme describes the emotional intensity of these experiences which may require additional support both for parents and practitioners. The final theme of The Ripples describes some parents’ shifts in understanding and compassion that extended beyond themselves and their infants. Together, the four themes reflect both positive and negative experiences, for parents and practitioners, alongside practical considerations for delivery within the PMHS context. Illustrative quotes have been condensed for readability and accompanied by parent identifiers (using A-I to indicate study site) or practitioner identifiers (using PRAC).

### Theme 1: Flamingos

*“Even though at home they were all on the screen, it was like I wasn’t alone and we were all in this together … … I used to call it my flamingo group … …because when female flamingos become mums, they lose their pink colour, because being a mum takes it out so much, and then when their baby flamingo grows up a little bit more, they get their pink back. So I said [to the group] it’s fine, we’re all going to get our pink back one day and even that sort of brought us all together. And we just felt like a group of friends.”* (B34 – naming quote)

This theme is named from an analogy (B34, above) which we interpreted as capturing the shared challenges, group dynamics and the power of the group. As indicated by the naming quote, for many, parenting demands were framed specifically as relating to motherhood; this reflects the gendered nature of the group, which was open to birthing parents and therefore predominantly women and predominantly primary caregivers. Additionally, in this study, all practitioners were women. Initially, many parents experienced apprehension about group participation yet ultimately found the format “better than expected” and sometimes perceiving this as preferable to individual intervention. The five sub-themes highlight diverse group dynamics, capturing both benefits and challenges.

#### 1.1 Demands of motherhood and parenting

Parents commonly described COS-P content, group peers, and practitioners as providing collective normalisation and validation regarding demands relating to parenthood and to mental health, particularly those endured as a mother or primary caregiver. This fostered reduced feelings of shame associated with parenting and mental health struggles, supported by feeling released from pressures through the COS-P principle of being a “good enough” parent and through reducing the feeling of being “alone” with challenging experiences.

*“I feel like [COS-P] gives you that support, the support and the understanding that … … you do know what you’re doing or you are good enough. It was, you know, nice to know how I was feeling, other people were feeling the same and I wasn’t alone.”* (B23)

Some parents valued practitioners’ personal parenting experiences, as fellow mothers, enhancing perceived understanding and connection:

*“She was a mum herself, it's always better when someone's leading the group who's gone through the baby thing already … … If you're talking about sleepless nights or bonding issues or whatever it is, I think if someone's not had a baby, they can't quite connect and fully understand what you're going through.”* (G27)

Although most parents valued COS-P as normalising the demands of motherhood and parenting, a minority found it amplified feelings of inadequacy, particularly through negative self-comparison with other group members. Practitioners shared similar insights about potential for negative self-comparison and emphasised the importance of considering individual suitability for group work, with one providing an example about interpersonal sensitivity.

Some parent participants reported that the group had led to improvements in their mental health, particularly where they experienced parenting demands and parenting-related stress as being deeply intertwined with their mental health:

*“It’s taught me to be more patient with [baby] too. … … I’ve just done everything I can to stop her crying, because I can’t deal with her crying. … … I now allow her to express herself, whether that’s good or whether that’s bad. And it’s made my anxiety so much more better because I know that actually, that it’s almost benefiting her.”* (A14)

However, others considered their mental health difficulties to be longstanding and beyond COS-P’s remit, limiting relevance of the flamingo analogy given the analogy’s focus on temporary loss of colour during early parenting:

“*I haven’t really changed my mental health problems because they’re very complex and they’re not going to change. I’ve been working on them for years now.*” (G10)

#### 1.2 Solidarity and seeking connection

Some parents deeply valued connecting with other group members, indicating this was a new and positive experience fostered by peer responses and support from practitioners. Some described feelings of personal pride and newfound confidence gained by stretching themselves into an initially uncomfortable group space.

*“I’m not the kind of person that has the confidence to be able to talk to people or to talk out. In the group I just had that great relief like okay I can talk.”* (G22)

However, other parents continued feeling isolated, either emotionally or socially, with some perceiving the online format (even with occasional face-to-face contact) as insufficient to meet their social connection needs for meeting with other mothers.

*“[online] you have your meeting, you watch the videos, you talk about the videos, and there's no have a cup of tea and a biscuit and a chit-chat”* (A23)

Group policies regarding external contact varied across sites. Some parent participants found group endings abrupt, wishing for continued interaction with group members.

#### 1.3 Small flocks and changing flocks

Group size and consistency was relevant for many participants in evaluating acceptability of COS-P. We drew on the flamingo analogy to frame this in similar terms, i.e. small flocks and changing flocks. Several groups had fewer than the intended 4–6 parents due to non-attendance. Generally, parents and practitioners appreciated hearing diverse experiences from other members and valued practitioners’ skill in supporting inclusive participation.

*“[Practitioner] would bring it back to the point in just a nice concise way. … … She would try to make sure everybody had an opportunity to share.”* (A23)

Some parents preferred smaller groups, which facilitated deeper sharing and closer connections; however, for some parents and practitioners, being part of a small group compromised acceptability. There were also examples where acceptability was compromised by a group being inconsistently attended (i.e. changing flocks). Here, challenges included feeling pressured to contribute or that the “group” aspect was fundamentally lacking.

*“We had quite varying attendance each time … … each session almost was a different group of people.”* (F37)

#### 1.4 Peers or not

Parents frequently valued participating with other mothers experiencing PMH difficulties and – as indicated in the naming quote – the aspects of shared identity accompanying this. However, others felt that differences existed between group members that compromised their comfort within the group and their comfort within conversations. Felt differences were articulated by parents and practitioners regarding trauma and loss (usually relating to childhood trauma and perinatal loss), and regarding current parent-infant relationship difficulties. Here, a bereaved parent describes her challenge about what to share within the group when not feeling like a peer:

*“[referring to perinatal loss prior to this baby] I might not have the same circumstances as all the other women on the call, so it might not be traumatic for them but it was traumatic for me in some ways. So, it was just how I expressed that without obviously upsetting anyone else.”* (I26)

In this example, a parent questioned her ‘right’ to be struggling with her mental health or to be accessing the group, when hearing about markedly different childhood experiences of group members:

*“I will say there were some points where I felt, I don’t know how to word it, but people mentioned a lot of their mental health things that would come up, or things that had happened in their childhood, and I don’t know if you can get Imposter Syndrome about having mental health issues, but I kind of felt almost like inferior. … … I feel like people had other things going on, and maybe what I was going through wasn’t, this says a lot more about me than the group I guess, that I didn’t have enough of a right to be there.”* (I13)

Practitioners identified that these perceived differences could intensify shame, highlighting the importance of assessing both individual and group suitability for COS-P- considerations that the trial design restricted compared to typical clinical practice, as illustrated in this practitioner quote about differences in the nature of group members’ parent-infant relationship difficulties.

*“[One] mum was experiencing quite difficult feelings towards her baby, everyone else was at a very different point. So, I think that felt quite isolating and, sort of, almost highlighted even further how bad and how shameful it was to have these thoughts and feelings. … … [in the trial] we’ve had no control over who goes into the group, you can’t really think about those dynamics of how the babies all are or what their risk picture might look like in relation to someone else’s. So, they’re other things that you might think about pre doing another group in terms of that readiness to be part of it.”* (PRAC13)

#### 1.5 Sociocultural norms and gendered perspectives

Some parents and practitioners articulated COS-P’s relevance for all caregivers and valued that videos and materials did not solely depict mothers. Participants also identified untapped opportunities to include partners within COS-P. Aligning with the flamingo analogy’s emphasis on it being a loss of colour amongst “female flamingos … because being a mum takes it out so much”, some expressed that inclusion of all caregivers was important both to extend learning to both parents (where applicable) and to challenge sociocultural norms about gendered responsibilities for parenting.

*“Any sort of perinatal support should be for both partners. … It's just silly that it's just one partner that leads the support. And then somehow they're gonna have the capacity to impart that knowledge to the other caregivers, it’s just crazy.”* (F15)

*“Why should the emotional wellbeing of the next generation of humans all rest on the shoulders of the mums?”* (PRAC08)

Practitioners critically reflected on the limited demographic diversity of trial groups, linking this explicitly to broader inequalities in accessing PMH services and having limited ability to comment on the extent to which COS-P may fit across sociocultural contexts. One practitioner specifically questioned COS-P’s suitability for collectivist cultural contexts:

*“Actually it [COS-P] seems to fit kind of very much kind of with individualistic cultures. And it's really made me think actually, how can we make this fit for kind of more collectivist cultures.”* (PRAC02)

### Theme 2: Practise Babies

*“I think you’ve just got to practise. I think that 30 per cent is good enough doesn’t make you give up at the first hurdle’cause you think you’re not doing it right enough and you’re not being peace, love and light all the time. … … my older [children], they’re the ones I feel the guiltiest about because they were my practise babies and I grew up with them. Just being able to try and make reparations has been massive for them. … … I’m not always successful, but they can see I’m trying, that I’m not perfect, and that’s okay.”* (F11 – naming quote)

This theme captures the universal necessity and benefit of practising relationship skills – both in parent-infant relationships and in other relationships – accompanied by the importance of having permission to be learners, without expectations of perfection and with opportunities for repair. While named from a quote of a parent’s reflection on the importance of practising and learning across children (here, including feelings of regret and seeking to make reparations), the theme more widely addresses the value and potential difficulties for parents and practitioners alike of applying relational learning across relationships and the role of time in practising such skills.

#### 2.1 All parents need practise

Some parents and practitioners highlighted the potential stigma associated with parent-infant relationship difficulties and with parenting programmes, leading some parents to feel conflicted about taking part, in feeling this may indicate having “failed” in some way:

*“When I first discussed about joining it, I wasn’t really keen on the fact that it was like, called a parenting course. Because I felt like it was implying that I didn’t know how to parent my child.”* (C31)

However, discomfort typically diminished through the COS-P content, group discussions and practitioner contributions normalising parenting challenges:

*“I love the kind of constant emphasis on good enough, and kind of normalising, the ‘no parent gets it right’ … I think that has really helped with women that were quite nervous about participating.”* (PRAC01)

Critically, this normalising was accompanied by a perspective that change is possible, and can be achieved through practise, where practising involves making and repairing mistakes, as conveyed in the theme’s naming quote and here:

*“That message of like, you know, it's not about being the perfect parent and everyone makes mistakes. Everyone's human. And it's about like sort of being aware of that and, you know, if mistakes do happen or you fall off the circle and then it's about, like, how to repair that relationship with your child and stuff.”* (H10)

Many parents described a supportive and non-judgemental programme environment, which appeared to foster their freedom and safety for learning and reflection. Nonetheless, some parents continued to struggle with self-criticism when practising:

*“A lot of the difficulties that I had with the course material is that…if I find, for whatever reason, that I am doing something ‘wrong’, inverted commas, I turn that in on myself. … … I use that to sort of bash myself and just kind of really guilt myself about things.”* (C12)

#### 2.2 Role of practise in COS-P

Many parents reported that practising skills between sessions reinforced their learning and improved their relationship with their baby (and potentially other family members, as indicated in the naming quote). This often involved reinterpreting their infant’s behaviours and cues, shifting their perceptions about their infant or how their infant perceived them, enhancing their understanding of their infant’s emotional needs, increasing their parental confidence, and – for some – reducing fears about their relationship with their infant.

*“I always felt like rejected and that we clearly didn’t have a bond because she loves my husband a lot. … … And that made me feel rejected but also that clearly I can’t do this [parenting] because she doesn’t even want me, and I felt unwanted. But because of [COS-P], I’ve realised that actually she does want me just as much. The circle is still there. I do see now that her cues are that she does want me.”* (C29)

While many spoke of feeling calmer, more patient and more confident as parents, some felt that COS-P did not fully equip them with the necessary emotion regulation skills, illustrated by this quote in describing challenges with practising between sessions:

*“When I’m frustrated and struggling, I don’t find myself automatically thinking, oh I need to apply this to the Circle and manage my feelings better. I’m not that kind of person. I really struggle to manage my own emotions, so I find it really hard to manage [baby’s] as well.”* (A36)

Critically, some parents believed that practising relationship and parenting skills was insufficient for addressing deeper issues of bonding or easing associated emotional distress, prompting them to seek additional interventions from PMHS:

*“I think it was more aimed at parenting rather than actually helping with* a *bond. … … it was all about behaviour [whereas] I wanted somebody to come along and go, ‘right, this is how you can learn how to find a bond with your child’, but the circle to me just seemed completely unrelated. … ….it just felt, like, a bit of wasted time when I could have potentially been doing other therapy.”* (H15)

*“I don't think this course would have helped [earlier] because even though I couldn't quite get that bond with him, I don't think this course would have taught me otherwise at the time. I think I was very much in the depression and nothing would have got me out of it until I was out of it.”* (F30)

#### 2.3 Comfort with (parenting) “practise” being observed

Parents generally valued observing other parents practising their parenting, both through COS-P videos and group members’ stories. Nonetheless, some found these videos emotionally challenging, particularly when experiencing significant PMH difficulties, highlighting the necessity of sensitive, facilitated discussion. The timing of COS-P in someone’s care could influence how content was received, with some experiencing as reassuring and others as overwhelming or intensifying feelings of shame:

*“I’d see, in the clips things that I’d do … … So, it was quite reassuring [whereas] if that was shown to me when [baby] was that [younger] age, it would have been like, and so what? Like, I’m too busy in my own head trying to cope with my own emotions and help my feelings.”* (C31)

Turning to how parents experienced being observed whilst practising their parenting, most valued mutual sharing in the group but some remained cautious about potential scrutiny, particularly in contexts where children’s social care services were involved or where compulsory participation was perceived. Although COS-P does not formally use individual video feedback, some practitioners and parents had spontaneous discussions of live parent-infant interactions, and the associated “practise”. Practitioners occasionally found these challenging due to uncertainties regarding appropriateness of discussing these within the group and – more practically – time management:

*“The majority of clients were with their babies. And I think when you see them with babies and like, a few things happened. … [Sometimes] we had a lovely bit of interaction … … you could almost use that as “this is a real life interaction rather than a video” and actually really use it to validate how well a client is doing in their relationship with their baby. Also, it worked the other way of, I had a client who left their video on and their baby was very distressed and [describes a more difficult interaction]. So it was about then broaching that when we came back together as a group, which I think was quite difficult.”* (PRAC10)

*“…often the liveness [of parent-infant interactions] helps … … but I never, it's always, I'm not quite sure whether that's something that I should be doing as a [practitioner]. That, ‘I wonder where your baby is now you know on the, on the, on the circle’. And balancing that with me getting through the material. Which I find hard.”* (PRAC04)

Notably, one parent specifically desired more personalised feedback regarding her parenting practise to reduce self-doubt. Comfort with having skills practise observed extended to practitioners too; for example, one practitioner suggested that future supervisory coaching would benefit from use of session recordings while another expressed discomfort with having received supervisory coaching within a group format.

#### 2.4 Practise babies and practise children

This sub-theme addresses parents’ cross-learning with different infants and children within and between families (as indicated in the naming quote), alongside the learning that parents hoped to take forward to their future relationships with their baby as a child. Some parents encountered difficulties with practise in interpreting and applying COS-P principles with very young infants. This was particularly evident amongst first-time parents who were unfamiliar with developmental stages of older children. Although certain COS-P materials were broadly helpful, including the extra materials provided in the perinatal adaptation, both parents and practitioners indicated a need for additional examples tailored to very young babies. Furthermore, some questioned the fundamental suitability of COS-P for parents of younger infants:

*“We have some babies who were tiny, kind of 4–6 weeks. … I think actually for younger babies, I'm not sure [COS-P] works as well as it does for older children.”* (PRAC02)

Parents valued learning through examples involving older children within their own families or observed in other group members' interactions. Notably, parents described bi-directional learning and practise across their children – using experiences with older children to better understand their infants, and vice versa. These reflections covered various contexts, including older children ranging from toddlers to teenagers, and those with additional needs:

*“When I started filling my toddler's [emotional] cup, I noticed he would go off for longer because I engaged with him for those 10 or 20 seconds. So I [have] seen instant gratification from putting that strategy in place. I don't think that was necessarily the case with the baby.”* (E13)

While reflecting on their older children, some parents expressed regret but maintained hope around opportunities for relational repair, as illustrated in the theme's naming quote (F11). Other parents viewed COS-P primarily as initiating practising now, in preparation for future interactions as their babies grow, rather than for immediate relational improvement.

*“And I'm hoping that in the future with the rupture and repair that I will be able to utilise that as he becomes a bit older and kind of test the boundaries a bit more.”* (A32)

Conversely, there were occasions where parents felt COS-P highlighted concerns about having already caused “irreparable damage”, making practise futile. Some parents and practitioners noted these concerns could be more related to the timing of COS-P delivery in relation to parents’ other PMH care and current psychological distress, rather than solely related to infant developmental stages. Practitioners attempted to offer reassurance by providing alternative perspectives or emphasising COS-P’s hopeful, compassionate aspects.

*“…women whose babies were closer to one (year) were saying, ‘Oh my God, I didn't know this before. I've ruined my baby. And I've done everything wrong.’ And now they just end up feeling rubbish, so [as a practitioner] there's a lot of … … kind of ‘being with’ and offering that ‘good enough’. And ‘it's never too late’ aspects.”* (PRAC02)

#### 2.5 Intergenerational practise babies

Insights into past experiences of being parented and transgenerational influences could profoundly affect parents. We interpreted these as examples where parents may view themselves as “practise babies”.

*“I need to show [my children] I can be a mum. And I can be a better mum than what mine was. … … It's made me realise [my children] do need cuddling; they do need love. And it's not okay to put the blame on them. It's not their fault. The fault is my own mum's and my stepdad's, beatings to the neglect and like… I've never harmed the kids but like I've neglected them emotionally and with their hospital or doctor's appointments. Like I didn't show them the love that they deserve. I just pushed them away.”* (I34)

Some found these insights inspirational, providing reassurance that intergenerational patterns were not inevitable, offering hope that change was possible through practise:

*“[COS-P] made me reflect on like my own childhood and like, maybe aspects of my childhood that I do want to keep and pass down to [name of baby]. And then there's other aspects of my childhood that I want to stop. … … it's made me want to try and work on myself to just sort of deal with them anxieties and not pass them [on].”* (H10)

#### 2.6 Practitioners’ practise babies (personal, work)

Practitioners sometimes brought personal parenting experiences into COS-P groups, using them intentionally to facilitate deeper engagement among parents; we viewed these as examples of practitioners’ encounters with ‘practise babies’ from their personal lives. This sharing differed from their usual clinical practice, and attitudes toward self-disclosure varied amongst practitioner participants. Regardless of explicit sharing, practitioners experienced reflective engagement about personal parenting histories and traumas. Two cases were noted where co-facilitators had not continued with subsequent COS-P groups and comments highlighted the importance of preparedness and supervision for lead practitioners and for co-facilitators.

*“…it was quite emotive for me because I had [PMH difficulties] … … and it also came up in supervision around maybe some relationship stuff with my mum … … it was a very different experience for me to say my stuff at work where normally I would keep it quite private. But it felt really, after that supervision, it felt amazing.”* (PRAC11)

Practitioners also brought “practise baby” experiences from their professional roles (as did some parents), applying prior parent-infant work knowledge and gaining competence through initial and early COS-P groups. They noted personal growth in confidence and relational responsiveness, transitioning from rigid, content-focused delivery to a more flexible, responsive approach as familiarity increased, with the potential for earlier groups to have been more akin to psychoeducation:

*“I was perhaps still quite nervy myself, delivering the group. So, I just don’t think I’d got any space to sort of observe their emotions, or their expectations … I don’t think I sort of noticed any of those things.”* (PRAC08)

Notably, practitioners identified parallels between the need for practise in their role in noticing and addressing group members’ needs and group members' need for practise in their roles with their children.

### Theme 3: The Dark Things

*“You get to hear from other people what they’re going through, which is a privileged position ‘cause you don’t, people don’t tell you the dark things.”* (F24 – naming quote)

This theme describes the emotional intensity for parents and practitioners in encountering and working with emotionally challenging material within COS-P. These “dark things” relate both to current and past relationships, including participants' own experiences of being parented. Within both participant groups (i.e. parents and practitioners) individuals varied significantly in their perceptions of the appropriateness of the COS-P group format for exploring these sensitive topics. As indicated in the naming quote, discussing these “dark things” within the group could be deeply valued, yet also demanding – what we chose to describe as a “toll” – sometimes requiring additional support outside of COS-P sessions.

#### 3.1 Emotional toll should not be underestimated

Some parents and practitioners contrasted COS-P with other parenting programmes, emphasising COS-P's greater emotional demands, particularly concerning reflections on personal experiences of being parented.

*“It’s like opening the Pandora box, but for yourself, and it will enable you to just take all the demons out and make them your friend … I thought it would be like, this is how you look after your child, this is how you just make them feel safe. But it was so deep.”* (J17)

A minority of parents suggested it was possible for participants to explore personal experiences and relationships superficially, without significant emotional distress or the necessity to “open up too much” (*I28*). Similarly, some practitioners noted that COS-P materials, particularly videos, helped sessions feel less personally exposing:

*“[having a video] takes people outside of themselves, but then allows them to reflect on themselves by looking back at the video. So it makes it less personal … … I think the video makes it quite practical as well, and normalising, like this is what we can see. … …. I think the manual’s really helpful, because it guides the [practitioner] to hold the boundaries, and keep the reflection on track.”* (PRAC07)

However, some parents felt inadequately prepared for the emotional intensity of COS-P, and that COS-P needs “a bit of a warning” (J15) about potentially triggering content related to past trauma. Even with adequate preparatory support, some parents and practitioners highlighted additional considerations such as managing emotional responses after sessions and the availability of alongside and supplementary support (further explored in 3.4–3.6).

#### 3.2 Toll as investment but not payable by everyone

Some parent and practitioner participants viewed the emotional intensity of COS-P as a worthwhile investment for parents—essential for achieving meaningful change rather than something to be avoided or compromising acceptability. For some parents, the emotional labour was considered manageable when adequately supported by practitioners within a safe group environment.

*“I did find that like, sometimes I were talking about, like, you know, especially stuff when I was growing up, and some stuff being a little bit more difficult to think about, it did like make me question … … do I continue if it’s making me feel this way? … … but obviously if they don’t bring it up, you’re never going to deal with it. And they did help me deal with it.”* (C33)

While many parents and practitioners endorsed COS-P’s wider availability within PMHS, some questioned its fundamental appropriateness for this clinical setting due to its emotional demands:

*“I understand where it comes from, and I totally understood the programme and what its intentions were, but I think some aspects of it can be quite triggering to people with mental health conditions when they’re just trying to get through day-to-day.”* (C31)

Individual suitability was also a critical consideration. Some parents and practitioners highlighted potential difficulties with COS-P for those currently experiencing high psychological distress or trauma symptoms. There were also occasional examples of parents finding it difficult to hear about others’ experiences without feeling able to offer them comfort. These concerns echoed earlier sentiments regarding feeling disconnected from certain shared experiences:

*“It does feel slightly odd being not physically with those people, so that when somebody was sharing something quite difficult and they were maybe upset, that you couldn't kind of do the normal things that you might do if they were in the room with you.”* (F17)

When practitioners identified contexts where COS-P (particularly in an online group format) could be unsuitable or destabilising, they emphasised that these would typically form part of suitability considerations and decisions about sequencing of care in usual practice, outside of a trial context. Example considerations included acute mental health symptoms, unresolved trauma, and limited time within the service (which could impact trust and support levels).

*“If [parents] were still quite unwell, actually it was too triggering for them, I think it actually destabilised some people I think, and it felt that people who were maybe a bit further away from being acutely unwell, were able to more reflect on it, whereas for other people. … … when they were still in it, they were just like, ‘this is making me feel worse’. And that felt really hard, actually, as a [practitioner], like, I think there were times where I was like, is this? I both, like, really loved the intervention, but also felt at times that like, is this working for our client group?”* (PRAC12)

Suitability considerations included implications for both the adults and their infants or other children who might be present:

*“How do we contain I suppose that distress outside of [COS-P]? We couldn't always contain it within [COS-P]… … is it ethical that we're delivering this programme to a client who's got a baby, who has very limited support but she's being triggered back to this, [previous perinatal] loss and who else is there for her?”* (PRAC10)

#### 3.3 Demands on practitioner should not be underestimated

Practitioners consistently highlighted the complexity when simultaneously balancing individual group members' needs and managing group dynamics, which could be particularly challenging in an online format and with parent-infant dyads. Parent participants provided many examples of effective management by practitioners.

*“If there was something that we didn’t understand, she would replay it for us, she would repeat it for us, she would make sure that we were okay. She knew that some parts of the programme were quite challenging for us, at certain different points, so what was challenging for me, may not have been challenging for somebody else, but what was challenging for them, may not have been for me. But she took on everybody’s emotions, like beared us all in mind.”* (A14)

In addition, some parents relayed instances where practitioners inadequately enforced group expectations or did not fully attend to such ruptures. These occasional examples included turn-taking, camera use, and presence of family members:

*“I did, obviously, struggle a lot to get my opinions across, like some people weren’t really that fond of other people’s opinions but it was alright.”* (D11)

*“We've got asked to do it in a private space, so no one else was [there]. And at one point someone was doing it in a room with their husband in the background, and that made me feel quite uncomfortable. … I felt like I followed the rules and made sure I was separate. It was just me and my baby.”* (A39)

Some practitioners expressed concerns that describing COS-P merely as “psychoeducational” failed to capture its therapeutic depth and the requisite skills and supervision necessary:

*“I think a lot of the [PMHS] team could pick up the manual for [COS-P], and have the videos and deliver what’s there in the manual … but I think it’s the really nuanced process stuff, like what’s happening in the room, how it feels in the room …”* (PRAC13)

“*It feels like it's a very therapeutic space for women to feel safe and feel comfortable actually to be able to share quite a lot of difficult information that maybe they wouldn't ordinarily share. … … And I think that actually if we can hold that group and build that kind of safe space and ‘be with’ our clients that that helps build that security really.”* (PRAC02)

Practitioners mostly valued their coaching from COS International (the intervention developers) and had wanted to be able to access it for longer, or ideally to receive clinical supervision from PMHS staff trained in COS-P, given the complexity and therapeutic depth they felt was involved in delivering it. Some practitioners viewed that the cognitive and emotional demands placed on them would be lessened by co-facilitators being able to receive comprehensive COS-P training and equivalent specialist supervision. They explained that, as their co-facilitators were not provided with the specialist training and supervision, the co-facilitator role had been largely limited to technical or practical support (e.g., managing video sharing, chat monitoring), rather than enabling shared therapeutic engagement during sessions and reflective space outside of sessions, or easing practical delivery considerations (e.g. scheduling 10 sessions around leave).

*“I think there’s a richness of having two [practitioners] that can both talk during the groups, it doesn’t have to just be one person talking throughout the whole group, the other [person] can pick up on things, and kind of add bits in that are really rich, and notice things in the group as well, that somebody that’s not trained might not notice. And then also the reflective stuff afterwards, is really helpful, if somebody knows the model.”* (PRAC11)

#### 3.4 Toll may be difficult to detect online

Even experienced practitioners encountered challenges in assessing emotional distress within an online group format. Constraints included limited visibility due to camera positioning, microphone usage, and small on-screen displays, which were often shared with infants:

*“You have to just be a lot more astute, more aware when it’s online.”* (PRAC13)

Participants noted examples of effective emotional support, where practitioners successfully recognised and addressed parents' emotional needs, occasionally aided by co-facilitators observing non-verbal cues. However, there were also examples where parents reported feeling unnoticed or burdened by the expectation to initiate individual support, or that taking up a practitioner’s invitation to stay on at the end of a session would be visible to other members and feel uncomfortable.

The nature of any initial face-to-face contact, such as a pre-group meeting or preliminary in-person session, could also influence the practitioner’s ability to detect emotional cues online, enhancing their overall responsiveness:

*“I think that [meeting in-person] created, as a [practitioner], it created a sense of safety, ‘cause I got a sense of things that I wouldn’t have picked up online, it was just like a feeling to sense where people are at, and how people move the rest of their bodies, not just their face, and you can pick things up and see that.”* (PRAC07)

#### 3.5 Toll may spill outside sessions

Parents and practitioners emphasised the significance of emotional strain both during and after sessions, and that some content was particularly challenging. Parents sometimes adjusted their participation levels within sessions to protect themselves emotionally afterward. This self-management could be evident to practitioners and group members, or subtle enough to go unnoticed:

*“The days where the groups are on and I really wasn't feeling good in myself, I probably share less just because it would then be less that I dragged up and therefore less to deal with after it. … … I sort of knew that I'd have to, you know, reorder it all and pack it all away after the session was done. And actually I had to do that whilst looking after a baby and on my own.”* (F15)

Some parents found it emotionally demanding to return to their home environment, whether this was returning solo with their baby or returning to an environment that was shared with other family members who had been thought about as part of COS-P. Impact outside of sessions was therefore relevant across diverse family compositions and living arrangements, both for thinking about the impact on the parent and other family members, including babies and older children and other adults.

*“…there was no kind of bed down period where I could settle with my thoughts before I could see [my parents]. [describes living very nearby] you can't avoid them without it looking like you're avoiding them and creating an atmosphere.”* (F18)

#### 3.6 Alongside support may be needed from PMHS and own network

While some parents found sufficient emotional containment within the COS-P group itself, others required additional formal support from COS-P providers or broader PMHS teams. This extra support helped participants feel safer during and outside sessions, and facilitated informed decisions about the suitability of continued participation in COS-P:

*“[Without having ‘extra sessions’ with my named practitioner] I feel like I’d have been very overwhelmed by my emotions, and I think I would have probably dropped out. Just because I don’t know how to process certain emotions, I tend to run.”* (C33)

*“I could see the, kind of, negative impact that it was having on me and I knew that I wasn’t strong enough to continue doing it each week. So, you know, in order to, kind of, protect myself and my children, I stepped back and said that I can’t continue. … … … I don’t think I would have been able to continue as long as I did if I didn’t have that additional support [from psychologist and mental health nurse that were external to COS-P and providing alongside support].”* (J15)

Parents sometimes accessed additional support through individual catch-up sessions after missed group meetings or through informal check-ins with COS-P providers or other PMHS team members. Occasionally, parents felt they needed to advocate for this extra support themselves. One practitioner spoke about proactively communicating with other clinical staff regarding group members likely to need additional emotional support related to trauma:

*“There was always feedback to either the care coordinator or the nursery nurse, whoever was involved, just giving them a bit of a summary of each session so that they could then offer support if need be to the women.”* (PRAC09)

Practitioners also acknowledged variability in the other treatment that parents were receiving alongside COS-P, and shared their perceptions of the variation in the level of emotional support being provided outside group sessions:

*“[Alongside COS-P] you could have a client with a high level of support and a client who actually was just attending [COS-P] and maybe having baby massage …. And actually who's holding them outside of that [COS-P] session, who's there for them, who's containing that distress?”* (PRAC10)

Beyond formal support from the PMHS, parents frequently cited family and friends as crucial sources of emotional and practical support, helping them manage emotional impacts, providing reassurance, or assisting with COS-P strategies. However, while some parents did not express needing additional informal support, others indicated having little to no support network available.

### Theme 4: The Ripples

This theme’s name adopts the word “ripples”, used by two parent participants, which illustrates how parents’ shifts in understanding brought by COS-P were not confined to their mental health or their parent-infant relationship (sub-theme 4.1) and were interacting with wider support provided by the service (sub-theme 4.2). Unlike the other three themes, this theme drew only on parent data. In our analysis meetings, we interrogated the place of this theme. We note its elements of overlap with other themes, which is considered acceptable within reflexive thematic analysis, and understand this as a cross-cutting theme where the ripples offer a meta-level insight about the shifts in understanding and perspective at play in all the themes.

#### 4.1 Across relationships

Parents consistently spoke of having new understandings about themselves and their babies; for some, these extended to their older children and to other adults, both within and outside their family. We interpreted these altered understandings as being characterised by compassion. There were many examples of self-compassion:

*“I feel like I’m a bit more kinder towards myself doing this, because I understand what’s occurred and where it’s come from.”* (C29)

We understood these as shaped by the psychoeducation content but also linked to the mutual compassion flowing within COS-P groups; flow which included compassion received from the practitioners and other group members and feeling compassionate towards other members and towards parents more widely.

*“I seem to be much more lenient and patient with mums in the supermarket when their children have a tantrum, for example.”* (G10)

As expressed here, the ripples could extend to understand any relational dynamics within the family:

*“I think that it just helps you to see it as a bigger picture, like a family as a whole, rather than just your relationship with your child. It’s kind of all the different relationships.”* (I13)

Most common were parents offering examples of increased understanding and empathy towards their babies and older children:

*“I am taking things less personally now. I used to think, ‘oh they’re mad, why are they mad at me’  … … I think you forget that when they’re babies that they have other stuff going on.”* (F11)

*“[Through COS-P] I had an understanding of what [older child] was going through and what I was going through, and we could communicate better, which we’d never been able to do before.”* (E22)

Some parents also reported ripples in relationships with their co-parents (current or former partners), including greater empathy for their experiences of being parented:

*“I take [my ex-partner’s] feelings into account a bit more because of the way he was brought up. So I’m a bit more understanding of why he’s the way he is.”* (I28)

Although many parents valued COS-P’s non-blaming stance, in creating ripples of understanding and compassion, the programme sometimes left parents with challenging thoughts and feelings toward their own caregivers, highlighting a support need:

*“The only negative I can think of is now I understand my mum a bit better in terms of possibly her background and her childhood. But it means I have to, sort of, be a bit more open to being sympathetic towards her.”* (F24)

#### 4.2 Across interventions

Parents described integrating COS-P insights with various psychological and parent-infant-focused interventions, such as dialectical behavioural therapy, compassion-focused therapy, baby massage, and video interaction guidance. These examples illustrate ripples of understanding flowing between COS-P and other PMHS interventions, in addition to the opportunity to practise skills across these therapeutic spaces:

*“The mindfulness and like the imagery and things like that that we did with the [compassion-focused therapy] fitted nicely … … It was nice to have almost these other skills to calm my mind before I then go into the situation of trying to fix things or go in with a screaming child, you know, without my head going crazy.”* (B23)

*“[video interaction guidance] helped me to know I’m not doing such a bad job, but I was still beating myself up about it … … so I think, sort of in tandem, like, sort of together they helped build up this relationship with [baby].”* (C25)

*“I could implement what I’d learnt in [COS-P] whilst I was doing [baby massage]. … … [COS-P] made me aware of, well, she’s looking for you, she wants you there, she’s out on the circle and you’re her hands.”* (I26)

## Discussion

The aim of this study was to explore the acceptability of COS-P from the perspectives of parents and practitioners within NHS community PMHS in England. Acceptability was high from the perspectives of both parents and practitioners under specific conditions: when COS-P was considered individually suitable and parents received adequate support during and between sessions, parents and practitioners highly valued the group programme and parents reported benefits for their mental health and psychological wellbeing, their relationships with them selves and with their babies. Aspects of parents’ positive experiences aligned with findings from previous COS-P literature, including increased maternal competence [16], Themes 1 and 2), opportunities to observe, be observed, reflect and learn [16], Theme 2, “Practise Babies”), and shifts in understanding or “lens” [28], multiple themes). The current study highlights (through Themes 2 and 4) that these shifts in understanding were not confined to them and their babies, but rather extended across other relationships, aligning with Helle et al. [16], who examined COS-P as an adjunctive psychotherapy, and with Butler et al. [6] in synthesising parent perspectives across parenting programmes. This suggests the value of future research on the implications of parent-infant interventions for longer-term relational dynamics. Additionally, the “ripples” (Theme 4) indicated potential cumulative gains when receiving COS-P alongside other care, highlighting the need for future research into the complexity of receiving multiple interventions. Nevertheless, while many experiences were positive, others were ambivalent or negative, raising crucial considerations regarding preparatory and supplementary support for both parents and practitioners.

### Importance of preparatory support for parents, and timing of COS-P

The findings emphasise the importance of preparation for psychological interventions and the significance of timing aligned with individual and family needs within a multi-disciplinary treatment plan. These insights were identified through the interviews and focus groups, where parents and practitioners reflected on how preparatory support facilitated being able to take part in COS-P sessions (e.g. Theme 3.1). Such preparatory support for COS-P should not only set realistic expectations about content and group processes through pre-group meetings but also provide sufficient time to build trusting relationships with practitioners, given the consistently identified significance of the group facilitator’s role in parenting programmes [6, 31].

Regarding intervention sequencing within broader PMH care plans, some parent and practitioner comments indicated that COS-P might be more effective after initially addressing aspects of maternal mental health (Theme 3.2). For example, self-criticism, fears, worries and assumptions about what other group members were thinking about each other were apparent in ambivalent and negative experiences. High self-criticism can impair therapeutic alliances, often resulting in poorer outcomes [24]. Given the inverse relationship between criticism and compassion, cultivating compassion - essential for parent-infant bonding and parenting - is critical, with higher self-compassion positively associated with improved relationships [11]. Compassion-focused therapy in PMHS demonstrates significant benefits in enhancing self- and other-focused compassion [23]. Indeed, compassion was central to the positive relational ‘ripples’ described by parents in this study (Theme 4), reflecting compassion-focused therapy’s emphasis on giving compassion to others, receiving compassion from others, and giving compassion to ourselves [13]. While some parents attributed these positive outcomes solely to COS-P, some parents and practitioners identified the necessity of additional interventions targeting compassion or emotional regulation to perform the reflective practise involved in COS-P. Collectively, these insights suggest prioritising maternal mental health (for example risk stabilisation and management) as a foundational step for subsequent parent-infant interventions like COS-P. Additionally, some participants considered COS-P less suitable for very young infants, warranting careful consideration of timing within treatment planning. These points are not intended to diminish the importance of providing parent-infant interventions within a critical period of development, or to imply that maternal mental health difficulties are something to first be fully resolved. Rather, they emphasise the importance of psychological formulation to underpin a thoughtful and sequenced approach to perinatal mental health treatment planning that aligns with parents’ personal goals and preferences, consistent with person-centre care [43] and the crucial role of trauma-informed care in the perinatal period [22].

### Importance of alongside support for parents

The need for alongside support from PMHS and the wider multi-disciplinary team was evident in participants’ reflections (Theme 3.6), suggesting that COS-P may not always be sufficient in isolation and highlighting the importance of wrap-around support to meet individual parent needs. This aligns with Maxwell et al.’s [28] findings in early parenting services that COS-P alone can be insufficient or inappropriate for certain parents. Within this study, alongside support came from COS-P practitioners outside of scheduled group sessions and from other service members. We note that this study did not formally capture the number and nature of additional support contacts that were provided in supporting parents to attend COS-P, highlighting the need for researchers and practitioners to consider the resource implications of delivering additional check-ins, conversations between members of the multi-disciplinary team, or wrap-around care. Examples in this study (Theme 3.4) indicated that challenges in identifying the need for further support can be intensified by online delivery formats, echoing Cook et al.’s [7] reflections on difficulties monitoring non-verbal cues and maintaining therapeutic attunement remotely. Additionally, parents noted the importance of informal support in their own networks was highlighted (Theme 3.6) in enabling them to tolerate COS-P's emotional demands, consistent with research advocating family-inclusive approaches in PMHS [12].

### Importance of support for staff

This study supports previous research by Maxwell et al. [28] in illustrating the complexity involved in practitioner roles, particularly concerning managing group processes (Theme 1) and the practitioner’s essential role in sustaining group cohesion (Themes 1 and 3). However, the current findings extend this understanding by highlighting practitioners' own support needs (Theme 3) and emphasising the fundamental value of practise (Theme 2). Specifically, practitioners expressed preferences for co-facilitators to also be trained in COS-P, aligning with British Psychological Society Perinatal Best Practice Guidance [30], particularly to manage cognitive load effectively when delivering interventions remotely. Additionally, this study underscored the emotional impact for practitioners who are facilitating psychologically intensive group interventions and necessity of practitioner wellbeing support, acknowledging the ‘parallel processes’ [9] experienced in group facilitation. These unconscious mirroring processes between parents, practitioners, and supervisors are crucial in supervision or coaching contexts as they affect practitioners’ emotional responses, their clinical practice, model adherence, and intervention efficacy [29]. Lastly, ensuring access to supervision from individuals trained in COS-P is a recommendation from this research with practitioners in the study emphasising this as critical. Indeed, the need for high-quality training and supervision has been identified across parenting programmes [6] and national guidelines on delivering psychological therapies within PMHS identify that modality-specific supervisions maximises model adherence and improves treatment outcomes [33], here, this would facilitate resolving dilemmas encountered during sessions, such as managing ‘live’ parent-infant interactions and practitioner self-disclosure.

### Study strengths and limitations

This study achieved strong information power by hearing a diverse range of perspectives, and from both parents and practitioners. Sampling strategies ensured inclusion of individuals with a range of attendance, ranging from one to all completed sessions. Whilst low attendance was not synonymous with low acceptability, ambivalent and negative experiences were more common amongst those with lower attendance and we believe that intentionally sampling by attendance helped to promote a diverse range of perspectives. Unfortunately, practitioner participation was limited to two-thirds of eligible practitioners, potentially omitting differing perspectives, and in hindsight, the exclusion of co-facilitators represents a significant gap. Additionally, a “baby blindspot” [35] was identified through the process of reflexivity, as we did not explore explicitly how babies themselves might experience the groups, particularly regarding their role as “practise babies.” Alongside the “baby blindspot” highlighting aspects of the infant experience that were not explicitly explored, we also recognise that certain dimensions of maternal subjectivity—specifically resilience and ambivalence—were not explicitly connected to our analysis. As discussed by Baraitser & Noack [1], maternal resilience involves the capacity to navigate the challenges of parenting while bearing ambivalent feelings about oneself and one’s child. Although aspects of maternal strength and coping are evident across the themes in our study, the construct of resilience and ambivalence was not foregrounded, representing a potential conceptual blindspot. Reflecting on this lens may offer further insight into parents’ engagement with COS-P, particularly in relation to navigating closeness and autonomy with their infants, as reflected in the “going out and coming in” on the COS-P circle, which is a central component of the intervention.

An additional limitation is the over-representation of White British parents in the trial, reflecting broader inequalities in accessing PMHS [21]. Consequently, the findings cannot fully address acceptability concerning ethnic diversity and intersectionality. Given insights on sociocultural norms, gendered perspectives, and broader calls for cultural competence in PMH care and parent-infant interventions [8, 44], further research addressing intersectional representation should be prioritised.

Our collective reflection as a team is that the robustness of our interpretations has been enhanced by being an interdisciplinary team and with strong involvement of lived experience co-researchers. For example, our discussions considered how evaluating COS-P within a randomised controlled trial context introduces factors that may influence acceptability—sometimes differing from how members of the wider team experienced delivery outside of the trial, whether as recipients or providers. Within the trial, practitioners could not apply typical clinical suitability considerations regarding timing and group dynamics, and parents similarly lacked involvement in treatment decisions, possibly differing from routine clinical practice. Additionally, some parents expressed altruistic motivations for trial participation rather than perceiving a direct need for COS-P, potentially reducing perceived stigma or burden associated with participation [40]. Conversely, some of the comments from our wider lived experience panel and from trial participants suggest that for some individuals, the broader demands of trial participation may have increased perceived burden.

Despite these limitations, the study provides valuable insights for practitioners across various contexts, especially timely given COS-P’s increased availability within PMHS despite limited previous evidence. These findings aim to inform ongoing practice developments and ensure comprehensive support for both parents and practitioners.

## Conclusions

COS-P has received substantial investment internationally, outpacing its evidence base. This study significantly contributes to the evidence specific to NHS PMHS in England, which support birthing parents (typically mothers) experiencing moderate-severe and complex PMH difficulties. While many participants reported positive experiences with COS-P, others expressed ambivalence or negativity, highlighting potential gaps in representation of parents experiencing greater difficulties. Key findings underscore the necessity of comprehensive preparatory and supplementary support for parents and practitioners, addressing practical considerations and the emotional demands involved. Effective assessment of individual suitability must be trauma-informed, carefully considering intervention timing and sequencing, while prioritising personal agency and choice. The research also emphasises the considerable skill, resources, and modality-specific training and supervision required to effectively facilitate group-based parent-infant interventions, with learnings transferable to other psychological interventions.

## Supplementary Information

Supplementary Material 1.

## Data Availability

The datasets generated and/or analysed during the current study are not publicly available. As specified in the protocol, this is because, whilst the names of places and people will have been removed, the combination of contextual information given by participants could compromise their anonymity if the transcripts were available in their entirety.

## Abbreviations

COS-P: Circle of Security Parenting intervention
COSI: Circle of Security Intervention trial
PMH: Perinatal mental health
PMHS: Perinatal mental health services
